# Cryptic Zika virus infections unmasked from suspected malaria cases in Northeastern Nigeria

**DOI:** 10.1371/journal.pone.0292350

**Published:** 2023-11-08

**Authors:** Marycelin Mandu Baba, Abubakar Ahmed, Samaila Yaga Jackson, Bamidele Soji Oderinde

**Affiliations:** 1 Department of Medical Laboratory Science, College of Medical Sciences, University of Maiduguri, Maiduguri, Nigeria; 2 Department of Medical Laboratory Science, Faculty of Allied Health Sciences, Bayero University, Kano, Nigeria; 3 Department of Mathematical Sciences, University of Maiduguri, Maiduguri, Nigeria; University of South Florida, UNITED STATES

## Abstract

**Introduction:**

Although environmental and human behavioral factors in countries with Zika virus (ZIKV) outbreaks are also common in Nigeria, such an outbreak has not yet been reported probably due to misdiagnosis. The atypical symptoms of malaria and ZIKV infections at the initial phase could leverage their misdiagnosis. This study randomly recruited 496 malaria-suspected patients who visited selected health institutions in Adamawa, Bauchi, and Borno states for malaria tests. These patients’ sera were analyzed for ZIKV antibodies using ELISA and plaque reduction neutralization tests (PRNT) at 90% endpoint. About 13.8% of Zika virus-neutralizing antibodies (nAb) did not cross-react with dengue, yellow fever, and West Nile viruses suggesting possible monotypic infections. However, 86% of the sera with ZIKV nAb also neutralized other related viruses at varied degrees: dengue viruses (60.7%), West Nile viruses (23.2%), yellow fever virus (7.1%) and 39.3% were co-infections with chikungunya viruses. Notably, the cross-reactions could also reflect co-infections as these viruses are also endemic in the country. The serum dilution that neutralized 90–100% ZIKV infectivity ranged from 1:8 to 1:128. Also, our findings suggest distinct protection against the ZIKV between different collection sites studied. As indicated by nAb, acute ZIKV infection was detected in 1.7% of IgM-positive patients while past infections occurred in 8.5% of IgM-negatives in the three states. In Borno State, 9.4% of IgG neutralized ZIKV denoting past infections while 13.5% were non-neutralizing IgM and IgG indicating other related virus infections. The age, gender, and occupation of the patients and ZIKV nAb were not significantly different. ZIKV nAb from samples collected within 1–7 days after the onset of symptoms was not significantly different from those of 7–10 days. A wider interval with the same techniques in this study may probably give better diagnostic outcomes. ZIKV nAb was significantly distinct among recipients and non-recipients of antibiotic/antimalaria treatments before seeking malaria tests. The inhibiting effect of these drugs on ZIKV infection progression may probably contribute to the absence of neurological disorders associated with the virus despite being endemic in the environment for several decades. Also, protection against ZIKV as marked by the nAb was different among the vaccinated and unvaccinated YF vaccine recipients. Thus, the YF vaccine may be a good alternative to the Zika vaccine in resource-constrained countries.

**Conclusion:**

The cryptic ZIKV infections underscore the need for differential diagnosis of malaria-suspected febrile patients for arboviruses, especially the Zika virus. The absence of systemic surveillance for the virus is worrisome because of its association with neurological disorders in newborns. Co-infections with other arboviruses may impact adversely on the management of these diseases individually.

## Introduction

Zika virus (ZIKV), a re-emerging virus infection was first isolated in 1947 from a rhesus macaque in the Zika forest of Uganda, *Aedes* (Stegomyia) *Africana* [1], and in humans in 1952 [2]. ZIKV has a positive-sense single-stranded RNA genome and belongs to the genus *Flavivirus* of the family Flaviviridae [3]. It is transmitted primarily by the bite of infected *Aedes* mosquitoes but transmission through sexual [4] perinatal [5] and blood transfusion routes [6] have been also reported. Human Zika epidemics occurred on the island of Yap (Micronesia) [7], Gabon [8], and Senegal [9] from 2007–2008. This was followed by a major outbreak in French Polynesia in 2013 [10], New Caledonia [11,] and Easter Island [11] in 2014 resulting in many imported cases worldwide. At the end of 2014, the pandemic exploded with the virus circulating in 26 countries by March 2016 [10]. Brazil was the worst hit with an estimated 1.5 million cases followed by Colombia with >25 000 suspected cases and Cape Verde with >7,000 suspected cases [12]. In 2016, 2,439 cases of ZIKV-associated genital syndrome were reported in 22 countries and territories in the Americas in addition to 532,000 suspected and 175,063 confirmed cases in 48 countries and territories [13]. The situation prompted WHO to declare the disease a Public Health Emergency with International Concern (PHEIC) in 2016 [14].

Overall, routine surveillance for endemic arboviruses in Nigeria {including dengue (DENV) [15–17], yellow fever virus (YFV) [18], West Nile virus (WNV) [19], chikungunya virus (CHIKV) [20, 21]} is presently lacking. With regard to ZIKV, Nigeria seems to remain in the dark despite evidence of its circulation in the country [22]. Very few human ZIKV cases were reported in 1954 [23], 1975 [24], 1983 [25], 2016 [26] and 2020 [27] in Nigeria. Notably, studies that used only ELISA IgM/IgG may not have reflected the true ZIKV infections due to high degree of cross-reactivity among Flaviviruses. However, the ability of PRNT to neutralize the infectivity of a particular virus is considered a mark of protection against reinfection [18, 28] Additionally, PRNT measures the quantity of the antibody in the sample as well as the effectiveness of the antibody on the virus [29]. Although cross reaction may still occur with PRNT, but the use of a higher stringent endpoint titer of 90% in PRNT provides greater species specificity than IgM/IgG [30, 31].

Nigeria is one of the 32 countries in Sub-Saharan Africa that account for almost 93% of malaria death worldwide [32]. There have been so many externally funded interventions programs, and strategies to eradicate malaria in the country. These programs leverage the affordability, availability and accessibility of malaria diagnostics and therapeutics in three tiers of health care facilities in Nigeria as opposed to viral infections. Notably, ZIKV and malaria parasites are spread through the bite of different species of mosquito vectors. However, the initial phase of ZIKV infections mimic malaria with respect to clinical symptoms. This situation allows misdiagnosis and mismanagement of the infections caused by both pathogens. The absence of appropriate diagnostics for differential diagnosis of febrile illness for arboviruses leverage underreporting, under recognition and underestimation. This situation allows persistent and uninterrupted circulation of the ZIKV with a possibility of facilitating the spillover events that may result in global ZIKV outbreaks. This study targeted ZIKV by investigating its silent circulation among malaria-suspected patients and estimating its burden in three north-eastern states of Nigeria considering its association with congenital neurological disorders especially microcephaly in newborns.

## Materials and methods

### Study area

**Fig 1:**
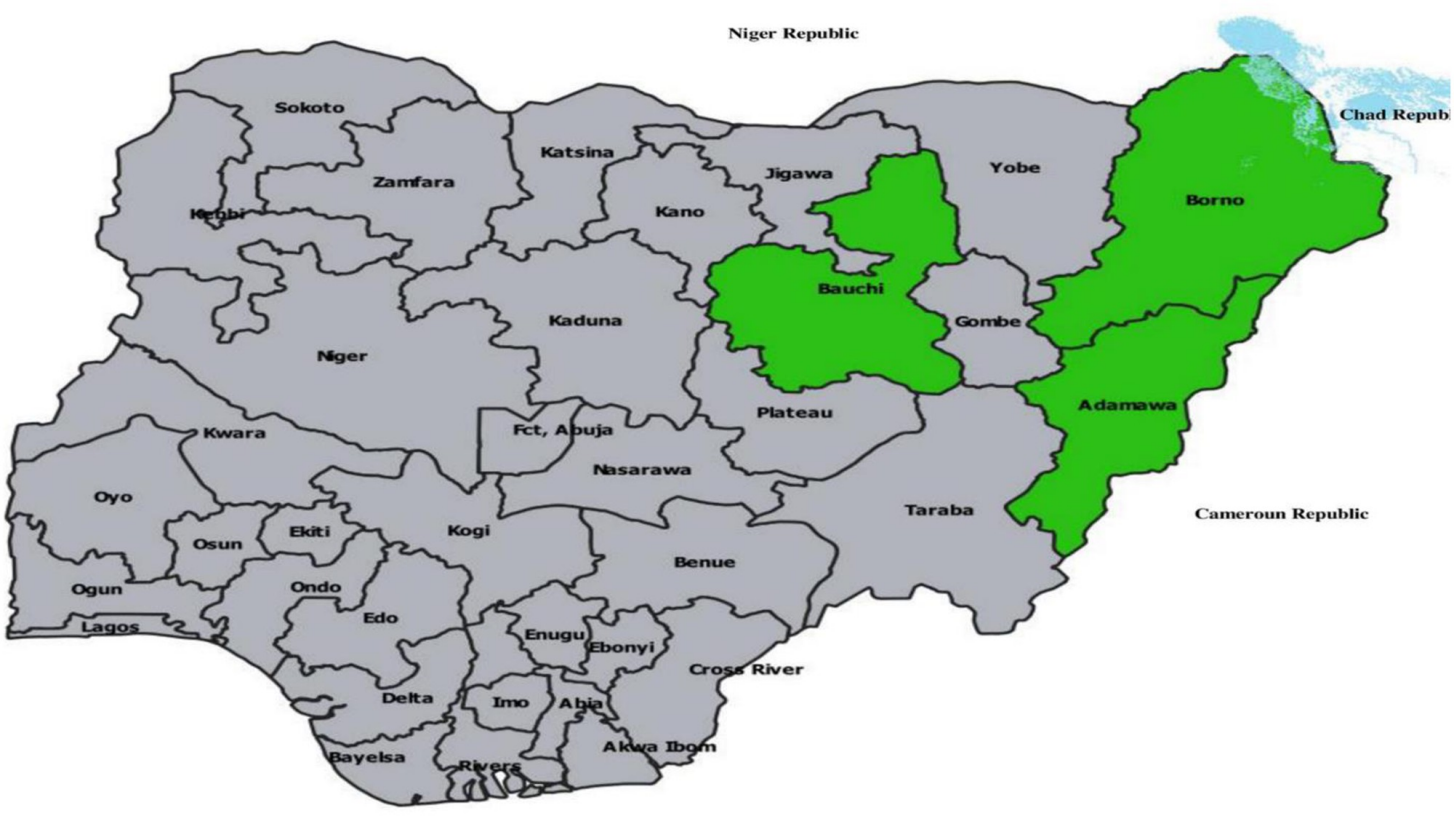
The map of Nigeria showing the sampling sites [16] under a CC BY license with permission from Mr. Kunle Ijala (2022), the Map producer.

Nigeria is located between latitude 9.081999 and longitude 8.67527 and covers an area of 923,769 square kilometers (356,669 square mi) with a projected population of 210,277,910 in 2022 [33]. North-eastern Nigeria is one of the six geopolitical zones and comprises six states (Fig 1). The simple random sampling technique was used to select three health institutions in three of the six northeastern states: Federal Medical Centre (FMC), Adamawa state, Abubakar Tafawa Balewa University Teaching Hospital (ATBUTH), Bauchi State and Specialist Hospital, Borno State (Fig 1). Adamawa state with an estimated population of 4.25 million in 2016 [34] has an annual average temperature of 35°C, sub-Sudan vegetation in the north, and Guinea savanna in the south. It is bordered by Borno to the northwest, Taraba to the southwest and Cameron to the East. Bauchi state has a population projected at 6,537,314 million in 2022 [35], a west Sudanian savanna vegetation in the south, a drier semi-desert Sahelian savanna in the north and an average annual temperature of 14°C to 38°C. Borno state shares border with Cameron to the East, Niger to the north and Chad to the northeast. It has an estimated population of 5. 86 million people in 2016 [34], and Sahel savanna vegetation in the north and Sudan savannah in the south with a daily temperature of 37°C.

### Population and sample collection

This study randomly recruited 496 patients with febrile illness who visited the selected hospitals to request a malaria test between April and August 2018 and endorsed the consent forms. A brief structured questionnaire was designed for each patient. Their socio-demographic characteristics and other parameters were collected using the structured questionnaire (Table 1). Although 200 serum samples were collected from each of the three states but only 96 samples from Bauchi state arrived the testing laboratory in good condition. Averagely 3 ml of each patient’s whole blood was collected by venipuncture into a serum separator vacutainer test tubes uniquely labeled for each consented participant. The blood was allowed to clot at room temperature for 20 minutes and afterward centrifuged at 3000 rpm for 5 min. The serum was thereafter aspirated into the sterile cryovials and stored at -20C until transported to the virology laboratory, the University of Maiduguri Teaching Hospital (UMTH). Qualitative IgM was performed for the five antigens (ZIKV, DENV, YFV, WNV and CHIKV) on samples collected from Adamawa and Borno States but samples from Bauchi was not sufficient in quantity for the five antigens. Also, qualitative IgG was performed for only Borno samples (200 of 496 samples) because of the high cost of the kits.

**Table 1:** Demographic characteristics of the febrile patients enrolled in the study.

Items	Adamawa	Bauchi	Borno	Total
**Sex**				
Female	104	54	108	266
Male	96	42	92	230
**Subtotal**	**200**	**96**	**200**	**496**
**Age (years)**				
**Age groups (years)**				
1–14	30	14	14	58
15–29	72	31	80	183
30–45	69	38	74	181
46–60	22	11	21	54
>60	7	2	11	20
**Subtotal**	**200**	**96**	**200**	**496**
**The interval between the onset of symptoms and sample collection (days)**				
**Interval (days)**				
1–7	147	54	169	370
7–10	52	33	31	116
7–14	1	0	0	1
Not specified	0	9	0	9
**Subtotal**	**200**	**96**	**200**	**496**
**Treatment with antibiotic/anti-malarial**				
**Treatment status**				
Treated	139	45	112	296
Not treated	59	42	88	189
Not specified	2	9	0	11
**Subtotal**	200	96	200	496
**YF vaccination status**				
**YF vaccination**				
Vaccinated	107	11	15	133
Not vaccinated	93	60	183	336
Not specified	0	25	2	27
**Subtotal**	**200**	**96**	**200**	**496**

### Sample size estimation

The sample size was estimated using the formula:

$$n\;\geq \frac{Z^{2}PQ}{e^{2}}$$

where *n* = sample size, *P* = prevalence, *Q* = 1 − *P*, *Z* = Z score at 95% Confidence intervals, *e* = margin of error. Using the past prevalence rate of ZIKV IgM of 6% (Mathe et al 2016) [26], the minimum estimated sample size was 87. However, assuming a prevalence rate of 50% in the population, the minimum sample size should be approximately 384. Therefore, we considered 496 to be a good representation of the population (29).

### Research ethics

The designed consent form was attached to the proposal submitted for ethical approval at each selected hospital. The research ethical clearance approval letters were obtained from the Research and Ethics Committee of ATBUTH (ref ATBUTH/ADM/42/Vol.1, dated 25 May 2017), State Specialist Hospital (SSH), Maiduguri, Borno State (ref no. SSH/GEN/641/Vol.1 dated 14 February 2018) and Federal Medical Centre, Yola (ref no FMC/YO/001/Vol.1 dated May 2018). The approved consent form was randomly given to 250 participants from each of the three states, to read, endorse and return if they agreed to be recruited for the study. Once minimum of 200 of the participants in each state endorsed the forms, we recruited them for the study. This include parents of children below five years who endorsed the forms on behalf of their children.

### Qualitative ZIKV IgM ELISA (Performed on Adamawa and Borno samples)

Sera samples were qualitatively analyzed for human ZIKV IgM antibody according to the manufacturer protocol (MyBioSource, San Diego, CA, USA). Briefly, 50ul of undiluted serum (positive, negative controls, and patients’ sera) was treated with 100 ul of HRP-conjugate and incubated for 1 h at 37°C. After appropriate washing, 50 ul of chromogen A and B were added to each well and incubated for 15 min at 37°C before the reaction was stopped. The plate was read at an optical density (OD) of 450 nm within five minutes after the reaction was stopped. The average of the positive control well was typically ≥1.0; the average of the negative control well was typically ≤0.15. The Cut-off values were the average of the negative control well + 0.15 in the samples tested. The cut-off OD units of ≥ 200 were considered positive.

### Qualitative ZIKV IgG ELISA (Performed only on Borno samples)

Sera samples were analyzed using Sandwich Complex ELISA kits (Diagnostic Automation/Cortez Diagnostics, Inc., USA, code: 811–35) according to the manufacturer’s protocol. Briefly, each diluted serum (1:40), including positive, and negative controls, and the patient’s serum was incubated with the ZIKV antigen in a coated microtiter plate. The plate was incubated at room temperature for 10 minutes. Enzyme conjugate was added to the wells after appropriate washings and incubated at room temperature for 10 min. The plates were washed and incubated with the chromogen at room temperature for 5 minutes before the termination of the reaction. The plate was read at an OD of 450 nm within one hour after the reaction was stopped. The cut-off OD units of ≥ 200 were considered positive.

### Plaque reduction neutralization test (PRNT) (Performed on all samples)

The PRNT was performed as previously described for the dengue virus with few modifications (36). Each serum sample was inactivated at 56°C for 30 minutes and stored at -80°C. BHK-21 cells were grown in Eagle’s Minimum Essential Medium (EMEM) supplemented with 10% fetal bovine serum, 2 mM glutamine, 2% Penicillin/Streptomycin, and 1% HEPES and 2.5% sodium bicarbonate. The cells were seeded in 24-well plates at a density of 1 x 10^5^ cells/ well and incubated at 37°C for 24–48 hours or until it reached 70–80% confluence. Each serum sample was diluted 1:8 to 1024 using in-house diluent (IHD) prepared with PBS supplemented with Penicillin/Streptomycin (22%), Gentamycin (0.,2%) and Fungizone (0.02%). An equal volume of ZIKV virus stock (obtained from Molecular Virology Laboratory, International Centre for Genetic Engineering and Biotechnology- ICGEB, Trieste, Italy) at a concentration of 100 PFU/ml was added to each of the diluted serum and incubated at 37°C for 1 hour. Each serum dilution–virus mixture was prepared in duplicate and three controls including a virus dose control (100 PFU virus plus diluent only), a cell control (diluent and cell only) and negative virus control were tested simultaneously. After incubation at 37°C for 1 hour, the virus-serum mixture was added to BHK-21 cells and incubated for 1 hour at 37°C. The plates were rocked gently every 15 minutes for uniform distribution of the inoculum. Different concentrations (10%, 12% and 14%) of overlay medium: Carboxyl methylcellulose (CMC) salt (Low viscosity) (Sigma) was tested using 100 PFU/ml of the virus to determine the best consistency. The best consistency of CMC was obtained at 6% concentration. Therefore, the overlay medium was prepared by mixing an equal volume of 12% CMC with EMEM supplemented with 4% fetal bovine serum (FBS) (After mixing both solutions, the concentration of the FBS in the overlay medium became 2% while that of CMC was 6%). 1 ml of the overlay medium was added to each of the 24 wells and incubated at 37°C for 10–14 days. Thereafter, each well was fixed with 500 ul of 10% formaldehyde solution for 30 minutes after removing the overlay and stained with 500 ul of 0.5% crystal violet (Sigma) for 20–30 minutes. The percentage of plaque reduction by the specific antibody was calculated using the formula: 100-(Number of plaques in sample/Number of plaques in control) x 100. The validity of the test was determined by the virus control having a minimum of 50 plaques and cell control and positive sera having no plaques at all. The PRNT titer was defined as the reciprocal of the serum dilution that reduced the number of plaques by 90% (PRNT_90_) [18, 36]. Modification in this study includes non-removal of inoculum before the addition of the overlay medium and the use of BHK-21 cell line instead of Vero unlike the previous report.

### Statistical analysis

The data was analyzed using Statistical Product and Service Solutions (SPSS), Version 25.0, from IBM SPSS. We used the mean standard deviation/standard error of the mean and percentage when appropriate for the patients’ characteristic description. Age groups and occupations were compared using the Pearson χ2 or Fisher’s exact test for categorical variables when the expected count is less than 5. Additionally, Kruskal-Wallis H test and Mann-Whiteney U test were used to determine the difference between ZIKV neutralizing antibodies and age, states, gender, treatment with antibiotics/antimalaria, urban/rural dwellers, intervals of sample collection and onset of symptoms and yellow fever vaccination status. Binary Logistic Regression in SPSS was used to obtain the odds ratios and confidence intervals for the association between ZIKV infection /immunity (PRNT_90_ results) and other variables in the three states. The fitness of the model to correctly predict the PRNT results (positive and negative) by the independent variables was tested and found significant using the Omnibus test of model coefficient and insignificant with the Hosmer and Lemeshow test. Overall, the percentage accuracy in the classification was 87% with 100% specificity (true negative) but 0% sensitivity (True positive).

## Results

### Demographic characteristics of the febrile patients enrolled in the study

Of 496 malaria-suspected patients studied, 230 were males while 266 were females in the three states as shown in Table 1. Their ages ranged from 2 to 86 years with a median age of 30 years. Other epidemiological variables analyzed include the interval between the onset of symptoms and sample collection, settlements (urban and rural) as well as their YF vaccination status.

### Distribution of ZIKV antibodies in three northeastern states of Nigeria

In Adamawa state, 15.5% and 14.5% in Borno had ZIKV IgM. However, 6 (3.0%), 35 (36.5%), and 24 (12.0%) in Adamawa, Bauchi and Borno respectively had ZIKV neutralizing antibody (nAb) by PRNT_90_. Overall, 31.1% nAb in the three states as shown in Table 2. Only 1 (1.7%) patient with ZIKV IgM also had nAb while 29 (8.5%) IgM negative were nAb positive (Table 2). Additionally, 170 (85%) of 200 patients in Borno State had ZIKV IgG and 16 (9.4%) of the IgG had ZIKV nAb while 23 (13.5%) had both IgM/ IgG. ZIKV nAb in the three states was significantly associated with Bauchi (χ^2^ = 57.01, df = 1, p< 0.001, Borno (χ^2^ = 0.36, df = 1, p = 0.590) and Adamawa (χ^2^ = 30.05, df = 4, p< 0.001) (Table 5). The significant difference between the three states was further confirmed using Krustal- Wallis H test (test-statistic = 63.102 (p< 0.001) (S2 Table). The odds ratio indicated that residents in Bauchi state are approximately 4 times more likely to acquire ZIKV infections as evidenced by the presence of antibody (Table 5).

**Table 2:** The distribution of Zika virus antibody in three states in Northeastern Nigeria.

State	No. Tested	ZIKV IgM	ZIKV PRNT	Total (+)
**Adamawa**	200	31 (15.5%)	6 (3.0%)	31 (15.5%)
*Bauchi	96	0 (0.0%)	35 (36.5%)	35 (36.5%)
**Borno**	200	29 (14.5%)	24 (12.0%)	53(26.5%)
**Total**	**496**	**60 (12.1%)**	**65 (31.1%)**	**119 (24.0%)**

About 9 samples at a dilution of 1:128 neutralized 90–100% of the virus infectivity while 16 did the same at 1:64, 13 at 1:32, 11 at 1:16, and 18: 1:8 (Tables 2 and 3).

**Table 3:** The titers of neutralizing antibodies to ZIKV in three northeastern states in, Nigeria.

States	Titers of neutralizing antibody to ZIKV						Total
	≤1:8	1:8	1: 16	1:32	1:64	1:128	
**Adamawa**	194	2	0	0	4	0	6
**Bauchi**	61	6	8	8	7	6	35
**Borno**	176	8	3	5	5	3	24
**Total**	**431**	**18**	**11**	**13**	**16**	**9**	**65**

### The cross-reactivity of ZIKV neutralizing antibody with other Flaviviruses

The sera of the patients were serologically tested for other Flaviviruses with the view to establishing their pre-exposure and cross reactions. Of 65 patients with ZIKV nAb (both acute and previous), 9 (13.8%) did not cross-react with other arboviruses tested while 56 (86%) did so at varying degrees: DENV (60.7%), WNV (23.2%) and YFV (7.1%). These cross-reactions are more likely to be co-infections. Similarly, 39.3% (22/56) of the patients were co-infections of Zika (a Flavivirus) and Chikungunya (an Alphavirus) viruses. (S1 Table).

### Distribution of Zika virus antibodies in rural and urban dwellers

The presence of ZIKV antibody was correlated with individual residence in the three states studied. The majority (84.3%) of the patients tested were urban dwellers while only 15.7% were rural. Among urban dwellers, 11.5% and 12.2% had ZIKV IgM and nAb respectively. Among the rural dwellers, 15.4% ZIKV IgM and 17.9% nAb were detected (Table 4). The type of settlement and ZIKV nAb were not significantly associated (χ^2^ = 1.16, df = 1, p = 0.180; OR = 1.35, Cl 95% = 0.79–1.44) (Table 5) and this result was further confirmed with Mann-Whitney U test (U = 15670.00, p = 0.357) (Table 5). However, there may be a need to reinforce surveillance for ZIKV in rural settings.

**Table 4:** Distribution of ZIKV antibody in rural and urban dwellers.

Type of Settlement	No. tested (%)	ZIKV infections	
		ZIKV IgM (%)	ZIKV nAb (PRNT_90_) (%)
Rural	78 (15.7)	12 (15.4)	14 (17.9)
Urban	418 (84.3)	48 (11.5)	51 (12.2)
**Total**	**496**	**60 (12.1)**	**65 (13.1)**

**Table 5:** Logistic Regression tables for all the independent variables.

Variables	β	Standard Error(β)	P-value (β)	χ^2^-value	P-value (χ^2^)	Odd Ratio (OR)	C.I Lower	C.I Upper
**State**								
Adamawa	2.1	0.44	<0.001	30.10	<0.001	0.21	0.095	0.44
Bauchi	1.95	0.29	<0.001	57.01	<0.001	3.81	2.75	5.26
Borno	-0.17	0.28	0.549	0.36	0.590	0.90	0.65	1.27
**Sex**								
Male	-0.34	0.27	0.217	1.53	0.229	0.83	0.59	1.14
Female (3–86 years)	-	-	-	-	-	1.16	0.93	1.44
Female (15–40 years)	-0.05	0.14	0.701	2.22	0.136	1.16	0.99	1.36
Others	-	-	-	-	-	0.58	0.27	1.25
**Settlement**								
Rural	-0.36	0.34	0.284	1.16	0.357	1.35	0.79	1.44
Urban	-	-	-	-	-	0.94	0.82	1.07
**Interval**								
1-7days	0.24	0.33	0.466	0.53	0.533	1.05	0.92	1.20
7-10days	-	-	-	-	-	0.83	0.49	1.39
**Treatment**								
Yes	-0.66	0.268	0.014	6.205	0.014	0.75	0.57	0.97
No	-	-	-	-	-	1.45	1.11	1.88
**YF-Vaccination**								
Yes	-0.89	0.38	0.016	6.08	0.015	0.49	0.26	0.91
No	-	-	-	-	-	1.20	1.07	1.35
**Age (Yrs)**								
1–14	-0.52	0.49	0.284	1.17	0.312	0.62	0.26	1.50
15–29	0.24	0.27	0.373	0.798	0.408	1.16	0.85	1.59
30–45	-0.08	0.28	0.776	0.08	0.785	0.95	0.67	1.35
46–60	0.007	0.43	0.986	<0.001	1.00	1.00	0.475	2.13
>60	0.162	0.64	0.801	0.064	0.737	1.17	0.35	3.88

### The age distribution of Zika virus antibodies among the patients studied

ZIKV IgM increased with increasing age up to 60 years and thereafter decreases. Overall, age was not significantly associated with ZIKV nAb (χ^2^ = 1.17, df = 7, p = 0.310; OR = 1.16, Cl 95% = 0.85–1.59) Table 5. With the Krustal-Wallis H test, no difference between the age and the ZIKV nAb was observed (test-statistic = 1.66, p = 0.800)(Table 5).

#### Gender distribution of Zika virus antibodies among patients in northeastern states, Nigeria

Among the males, 12.2% had ZIKV IgM while 11.7% were nAb. The distribution of ZIKV antibodies was categorized into females aged 3–86 years (Category 1) and those at child bearing age of 15–40 years (Category 2). In category 1, 9.8% were IgM and 14.2% had nAb. However, in category 2, 16.2% had ZIKV nAb. Among males aged 15–40 years, 10.7% had ZIKV nAb (Fig 3). Overall, gender (χ^2^ = 1.53, df = 1, p = 0.140; OR = 1.16, Cl 95% = 0.93–1.44) and occupation (χ^2^ = 6.40, df = 7, p = 0.450) were not significantly associated with ZIKV nAb (S1 Table).

### Distribution of Zika virus antibodies according to the interval within which samples were collected after the onset of symptoms

Of 371 samples collected 1–7 days after the onset of symptoms, 11.9% and 13.5% had ZIKV IgM and nAb respectively. Similarly, 116 samples collected 7–10 days were 13.8% IgM and 11.2% nAb (Table 6). ZIKV nAb and the interval within which samples were collected after the onset of symptoms were not significantly associated (χ^2^ = 0.53, df = 1, p = 0.290; OR = 1.05, Cl 95% = 0.92–1.20). This observation was also confirmed using Mann-Whitney U test (test-statistic = 21593.00,p = 0. 533) (Table 5).

**Table 6:** Distribution of ZIKV antibody according to the interval within which samples were collected after the onset of symptoms.

Interval (days)	No. tested (%)	ZIKV infections	
		ZIKV IgM (%)	PRNT_90_ (ZIKV nAb) (%)
1–7	371 (74.8)	44 (11.9)	50 (13.5)
7–10	116 (23.4)	16 (13.8)	13 (11.2)
Not specified	9 (1.8)	0 (0)	2 (22.2)
**Total**	**496**	**60 (12.1)**	**65 (13.1)**

#### Distribution of Zika virus antibodies among patients who received antimalaria/antibiotic treatments

The majority (59.7%) of the patients received treatment with either antimalaria and/ or antibiotics before seeking a laboratory test for malaria parasites. Among the treated, 14.2% had ZIKV IgM and 10.8% were nAb. Similarly, 9.5% and 16.4% of untreated had ZIKV IgM and nAb respectively (Table 7). Treatment with antimalaria/antibiotics was significantly associated with ZIKV nAb (χ^2^ = 6.21, df = 1, p = 0.014; OR = 1.45, Cl = 1.11 to 1.88) (Table 5). This difference was confirmed with Mann-Whitney U (test-statistic = 29925.00, p = 0.014) (S2 Table).

**Table 7:** Distribution of ZIKV antibodies among patients who received antimalaria/antibiotic treatments.

Treatment status	Total tested (%)	ZIKV infections	
		ZIKV IgM (%)	ZIKVnAb by PRNT_90_ (%)
Treated	296 (59.7)	42 (14.2)	32 (10.8)
Not treated	189 (38.1)	18 (9.5)	31 (16.4)
Not specified	11(2.2)	0	2 (18.2)
**Total**	**496**	**60 (10.9)**	**65 (13.1**

### Yellow fever vaccination status of patients and the distribution of Zika virus antibodies

YF vaccination status in Adamawa state was higher (53.5%) than in Bauchi (25.0%) and Borno (7.5%) (S1 Table). Overall, only 26.4% of the patients tested received the YF vaccine in the three states. ZIKV IgM was detected among 31.7% of the vaccinated and 6.3% of the unvaccinated. Only 13.8% of the vaccinated as against 15.3% of the unvaccinated had ZIKV nAb (Fig 3). ZIKV nAb was significantly associated with the YF vaccination status (χ^2^ = 6.01, df = 1 p = 0.015). The difference was further confirmed with Mann- Whitney U test (test-statistic = 21882.00, p = 0.15) (Table 5).

## Discussion

The results of this study have demonstrated the misdiagnosis of ZIKV infections for malaria in Nigeria and underscore the importance of screening malaria-suspected cases for arboviruses.

To estimate the burden of ZIKV in Nigeria, both ELISA IgM/IgG and PRNT were used.

In this study, 13.1% had ZIKV nAb which indicated both acute and past infections but only 1 (1.7%) IgM in the three states had nAb, indicating acute infection while 11.9% were infections by other flaviviruses (S1 Table). In Borno state, 9.4% of ZIKV IgG also had nAb indicating past ZIKV infections. However, the persistence of ZIKV IgM up to 12–19 months after illness onset confounds the recent and past infections concept [37]. Furthermore, the ability of high antibody titre (1: 64–1: 128) to neutralize the virus at 90–100% (Tables 2 and 3) is highly suggestive of ZIKV (S1 Table) in agreement with a previous report [31]. However, the interpretation of PRNT_90_ positivity with high antibody titre amidst co-infections may be confounded by the concept of ‘original antigenic sin’ (antibody to the earlier virus may be higher than the new) [38]. Additionally, 13.8% of ZIKV nAb did not cross-react with other flaviviruses indicating possible monotypic infections while 86.0% did so with DENV, WNV, and YFV in agreement with previous reports [39, 40]. These cross-reactions may be co-infections since these viruses are also endemic in the country. Our speculation is supported by a report that the presence of co-circulating arboviruses increases the chance of coinfection and demonstrates the importance of differential diagnosis [40]. Similarly, co-infections of Zika and Chikungunya viruses were observed in 39% of the patients studied in agreement with previous reports [41–43]. Overall, arbovirus co-infections observed in this study agreed favorably with previous reports [44–47]. This is possible because Aedes aegypti and Aedes albopictus (vectors of DENV, CHIKV, YFV and ZIKV) are capable of transmitting multiple arboviruses during a bite [45]. Thus, these arboviruses have common hosts (humans), mosquito vectors, biological, ecological and economic factors [48] resulting in epidemiological synergy, seasonality and attack rates [45]. We speculate that co-infections with other arboviruses may impact adversely on the management of these diseases individually.

**Fig 2:**
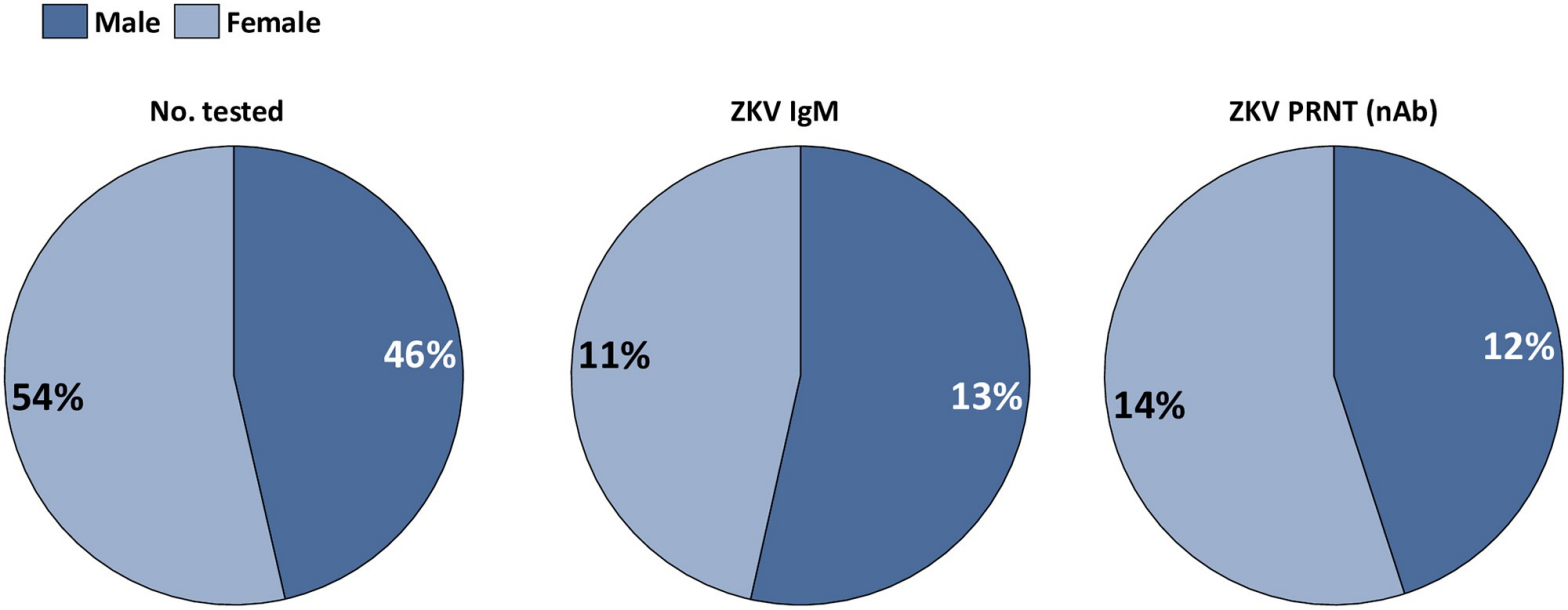
Gender distribution of Zika virus antibodies among patients in northeastern states, Nigeria.

ZIKV infections based on nAb were significantly higher in Bauchi than in Borno but least in Adamawa states (Tables 2 and 3). The three states differ in terms of vegetation and climatic conditions which impact vector competence and transmission of arboviruses as previously reported [49, 50]. We speculate that, in addition to the above-mentioned factors, genetic differences in the vectors/ human hosts, poor waste management, and hygiene practices in Bauchi favor more transmission of ZIKV than in Adamawa and Borno states. The distribution of ZIKV infections and settlements of the patients in the three states were not significantly different in contrast to a previous report where vectors of ZIKV mostly colonized urban areas [51]. Overall, urgent improvement on vector control measures, waste management and environmental sanitation including drainage system is necessary in the three states especially the rural areas with a high probability of sylvatic transmission due to intense agricultural practices. ZIKV infections and gender (Fig 2) were not significantly associated (χ^2^ = 1.53, df = 1, p = 0.229). However, 16.2% of females at childbearing age of 15–40 years having ZIKV nAb is alarming. The potential of ZIKV to cause stillbirth and other neurological disorders in newborn necessitates active surveillance especially in females of childbearing age. ZIKV nAb from samples collected between 1–7 days and 7–10 days after the onset of symptoms were not significantly associated (Table 6). The timing of sample collection and the purpose of the testing depends on the choice of assay. The use of a wider intervals with the same techniques used in this study may give a better insight into the diagnostic outcomes.

A significant number of the patients (59.7%) tested received treatment with antibiotics/anti-malaria before the malaria parasites tests. This practice is common in the country because every febrile illness is presumed and immediately treated as malaria and or bacterial infections. In this study, ZIKV nAb and treatment with antibiotics/antimalaria (Table 7) were significantly associated (χ^2^ = 6.21 df = 1, p = 0.014) and the untreated had more ZIKV nAb than those treated (OR = 1.45, Cl 95% = 1.11–1.88). This observation corroborated a report that some antibiotics (ivermectin, erythromycin, tetracycline) and antimalarial (mefloquine, chloroquine) are effective treatments for many viral diseases [52] including YFV and ZIKV [53]. We speculate that the inhibiting effect of these drugs on ZIKV infection progression may be contributory to the absence of neurological disorders associated with the virus despite being endemic in the environment for several decades. However, the effect of these drugs on ZIKV was not investigated in the current study.

**Fig 3:**
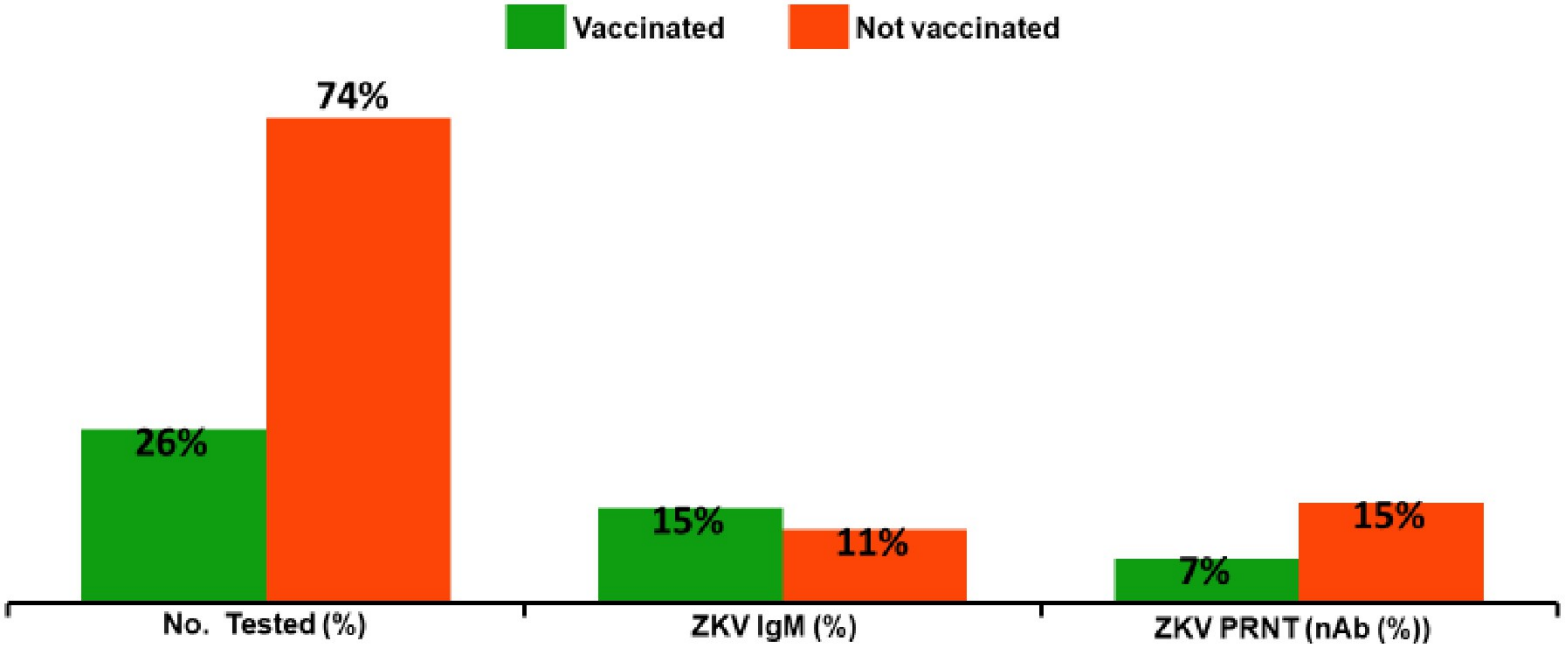
Distribution of ZIKV antibody according to the YF vaccination status of suspected malaria patients in northeastern Nigeria.

The low population immunity against YFV as evidenced by 73.5% of patients who did not receive the YF vaccine explains its periodic epidemics in the country (Fig 3). In this study, a significant association (χ^2^ = 6.08, df = 1 p = 0.015) between YF vaccine recipients and ZIKV nAb was observed. Our findings corroborated a previous study that demonstrated significant ZIKV–related microcephaly in the Northeast region of Brazil where YF vaccination coverage was the lowest [54]. The strong protection of YF vaccine against ZIKV in mice [55] has opened way for the use of YF vaccine for large-scale immunization during a ZIKV outbreak. Additionally, the significant immune response obtained from the use of a chimeric ZIKV vaccine developed with the 17-D YFV vaccine as the backbone [56] has provided a sound basis for the future development of ZIKV vaccine candidate. Meanwhile, YF vaccine may serve as a good alternative against ZIKV in resource-constrained countries.

The limitations of this study include our inability to test the samples from Bauchi and Adamawa for IgG due to the high cost of these kits. Also, PRNT was performed on ZIKV-positive sera diluted 1:8–1024 while other co-infecting flaviviruses were not diluted beyond 1:8 due to insufficient quantity of the samples.

### Conclusion

ELISA IgM and PRNT were used to unmask ZIKV infections from malaria-suspected febrile patients in Adamawa, Bauchi and Borno states at different prevalence rates. ZIKV IgM, IgG, and PRNT performed only on Borno samples allowed the detection of both acute and past infections. The species specificity of PRNT_90_ allowed the detection of true ZIKV infections. Co-infections with other arboviruses (DENV, YFV, WNV, and CHIKV) may confound the management of these diseases individually. ZIKV infections in females at childbearing age are warning signs for congenital Zika syndrome. Treatment with anti-malaria/antibiotics may ameliorate recovery from ZIKV. ZIKV nAb among samples collected within 1–7 and 7–10 days were not significantly different. YF vaccination may cross-protect against ZIKV. This study underscores the need for differential diagnosis of suspected malaria febrile patients for arboviruses, especially Zika virus. More affordable diagnostics in the market will ultimately facilitate the timely detection of these viruses before they spread, cost lives, and become difficult to control in resource-constrained countries.

## Supporting information

S1 Table The dependent and independent variables used in the analysis and interpretation of data on Cryptic Zika virus infections in Nigeria. (XLSX)

S2 Table Comparison between states, ages with PRNT results using Kruskal-Wallis H test. (DOCX)

S3 Table Comparison between two variables with PRNT results using Mann-Whitney U Test. (DOCX)

S1 Fig A microtiter well-showing plaques caused by ZIKV in a patient from Adamawa State. (JPG)

S2 Fig A microtiter well-showing plaques caused by ZIKV in a patient from Bauchi state. (BMP)

S3 Fig A microtiter well-showing absence of plaque—100% neutralization. (BMP)

S4 Fig A microtiter well-showing plaques caused by ZIKV in a patient from Borno state. (JPG)

S5 Fig A microtiter well-showing uncountable plaques- absence of neutralization. (JPG)

## Abbreviations

ATBUTH: Abubakar Tafawa Balewa University of Maiduguri Teaching Hospital, Bauchi
CHKV: Chikungunya virus
DENV: Dengue virus
ELISA: Enzyme-Linked Immunosorbent assay
EMEM: Earle’s Minimum Essential Medium
FMC: Federal Medical Center
ICGEB: International Center for Genetic Engineering and Biotechnology
IDP: Internally Displaced persons
IgG: Immunoglobulin G antibody
IgM: Immunoglobulin M antibody
nAb: Neutralizing antibody
PAHO: Pan American Health Organization
PRNT: Plaque reduction neutralizing antibody test
SSH: Specialist State Hospital
UMTH: University of Maiduguri Teaching Hospital
WHO: World Health Organization
WNV: West Nile virus
YFV: Yellow Fever Virus
ZIKV: Zika virus
