# Supplementary material for: Cryptic Zika virus infections unmasked from suspected malaria cases in Northeastern Nigeria
Source: PLoS One. 2023 Nov 8;18(11):e0292350. doi: 10.1371/journal.pone.0292350 (PMC10631648; doi:10.1371/journal.pone.0292350)
Supplement: S2 Table — (DOCX) [file pone.0292350.s002.docx]

**S 2 Table: Comparison between States, Ages with PRNT results using Kruskal-Wallis H test**

| **Variables** | **Kruskal-Wallis H Statistic** | **Mean Rank** | **P-value** | **Remark** |
| --- | --- | --- | --- | --- |
| **State** |  |  |  |  |
| Adamawa | 63.102 | 274.05 | <0.001 | Significant |
| Borno |  | 251.68 |  |  |
| Bauchi |  | 191.84 |  |  |
| **Age** |  |  |  |  |
| 1-14 | 1.66 | 259.62 | 0.800 | Not Significant |
| 15-29 |  | 244.01 |  |  |
| 30-45 |  | 250.00 |  |  |
| 46-60 |  | 248.25 |  |  |
| >60 |  | 243.80 |  |  |
