# Supplementary material for: Cryptic Zika virus infections unmasked from suspected malaria cases in Northeastern Nigeria
Source: PLoS One. 2023 Nov 8;18(11):e0292350. doi: 10.1371/journal.pone.0292350 (PMC10631648; doi:10.1371/journal.pone.0292350)
Supplement: S3 Table — (DOCX) [file pone.0292350.s003.docx]

**S 3 Table: Comparison of two variables with PRNT results using Mann-Whitney U Test**

| **Variables** | **Mann-Whitney U (Statistic)** | **Mean Rank** | **P-value** | **Remark** |
| --- | --- | --- | --- | --- |
| **Gender** |  |  |  |  |
| Male | 29925.00 | 252.45 | 0.392 | Not Significant |
| Female |  | 246.00 |  |  |
| **Yellow Fever Vaccination** |  |  |  |  |
| Yes | 21882.00 | 263.96 | 0.015 | Significant |
| No |  | 242.95 |  |  |
| **Settlement** |  |  |  |  |
| Urban | 15670.00 | 249.24 | 0.357 | Not Significant |
| Rural |  | 238.45 |  |  |
| **Treatment** |  |  |  |  |
| Yes | 26813 | 255.88 | 0.014 | Significant |
| No |  | 236.62 |  |  |
| **Interval** |  |  |  |  |
| 1-7days | 21593.00 | 246.97 | 0.533 | Not Significant |
| 7-10days |  | 253.44 |  |  |

**S 2 Table: Comparison between PRNT results using Mann-Whitney U Test for two independent groups Kruskal-Wallis H test and Mann-Whitney**
