# Supplementary figures and images for: Cryptic Zika virus infections unmasked from suspected malaria cases in Northeastern Nigeria
Source: PLoS One. 2023 Nov 8;18(11):e0292350. doi: 10.1371/journal.pone.0292350 (PMC10631648; doi:10.1371/journal.pone.0292350)

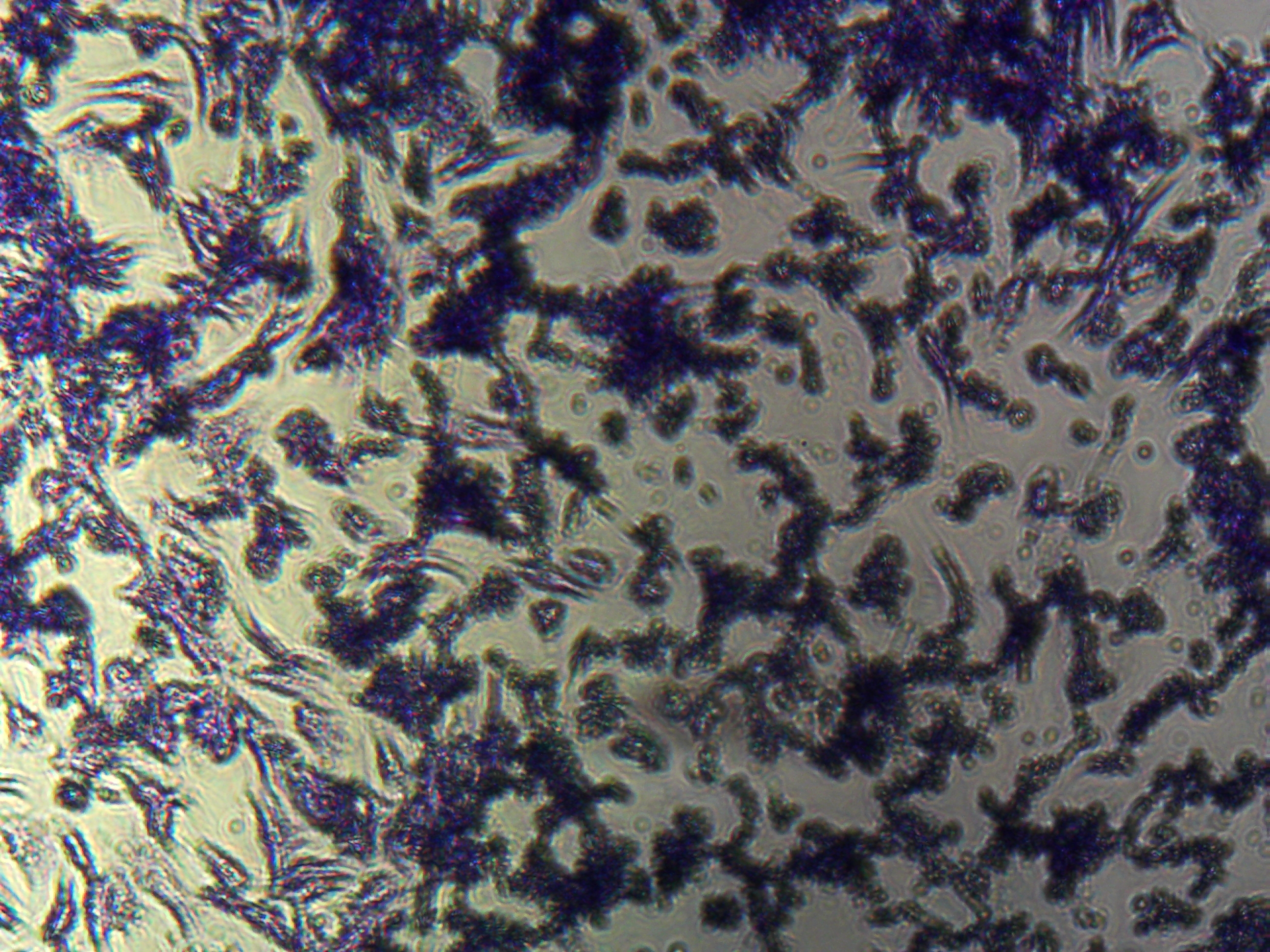

Supplement: S1 Fig — (JPG) [file pone.0292350.s004.jpg]

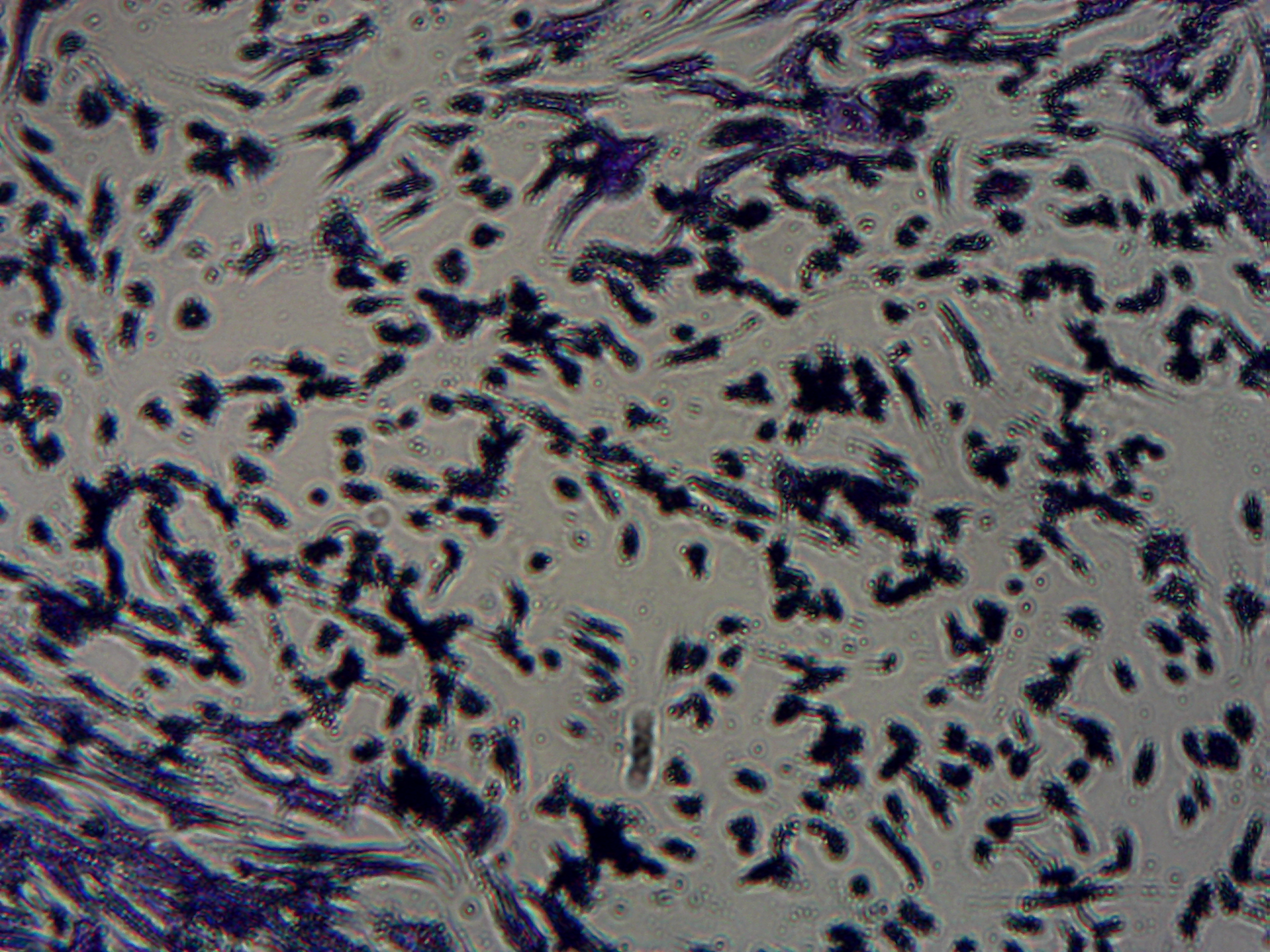

Supplement: S2 Fig — (BMP) [file pone.0292350.s005.bmp]

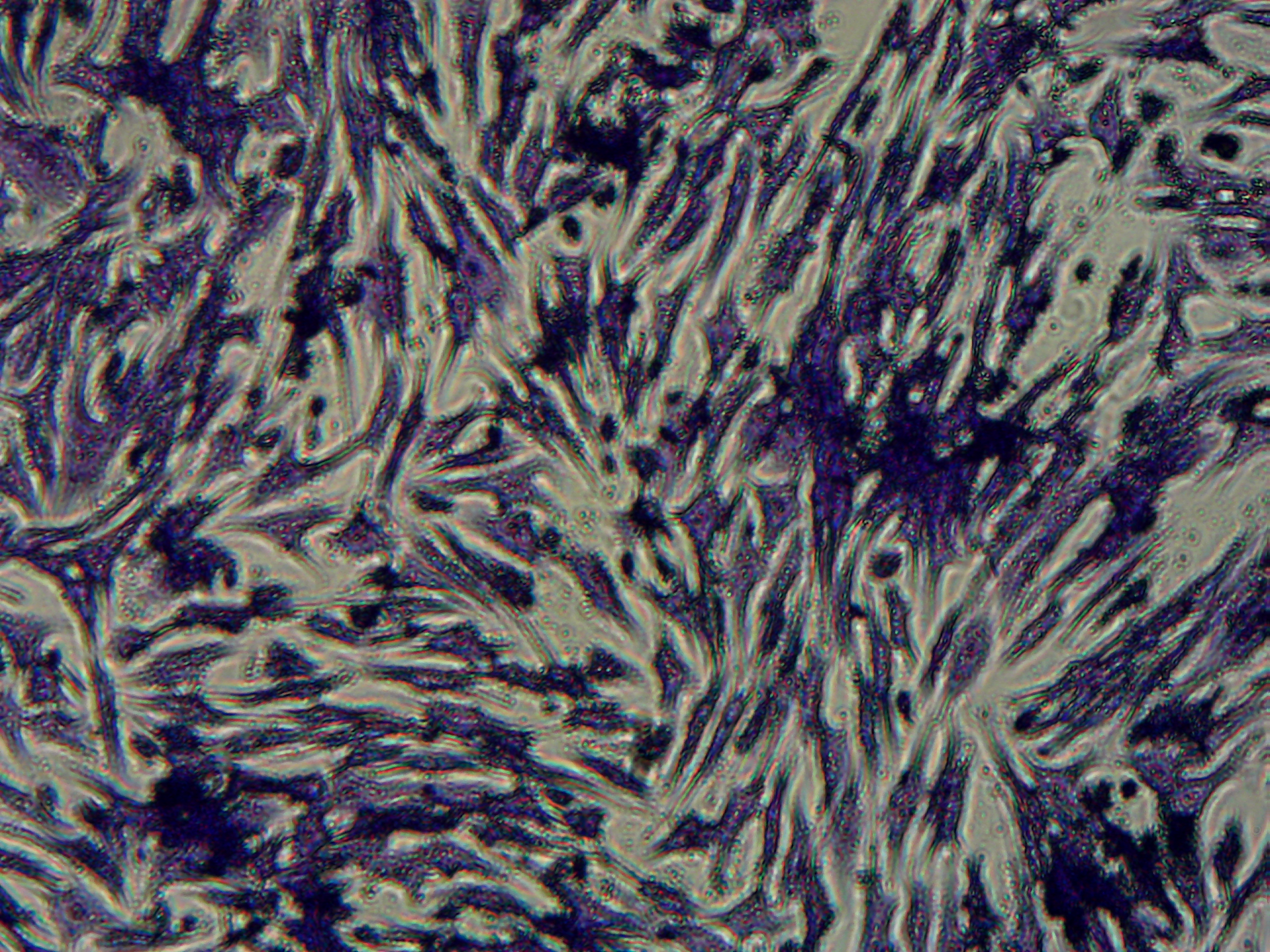

Supplement: S3 Fig — (BMP) [file pone.0292350.s006.bmp]

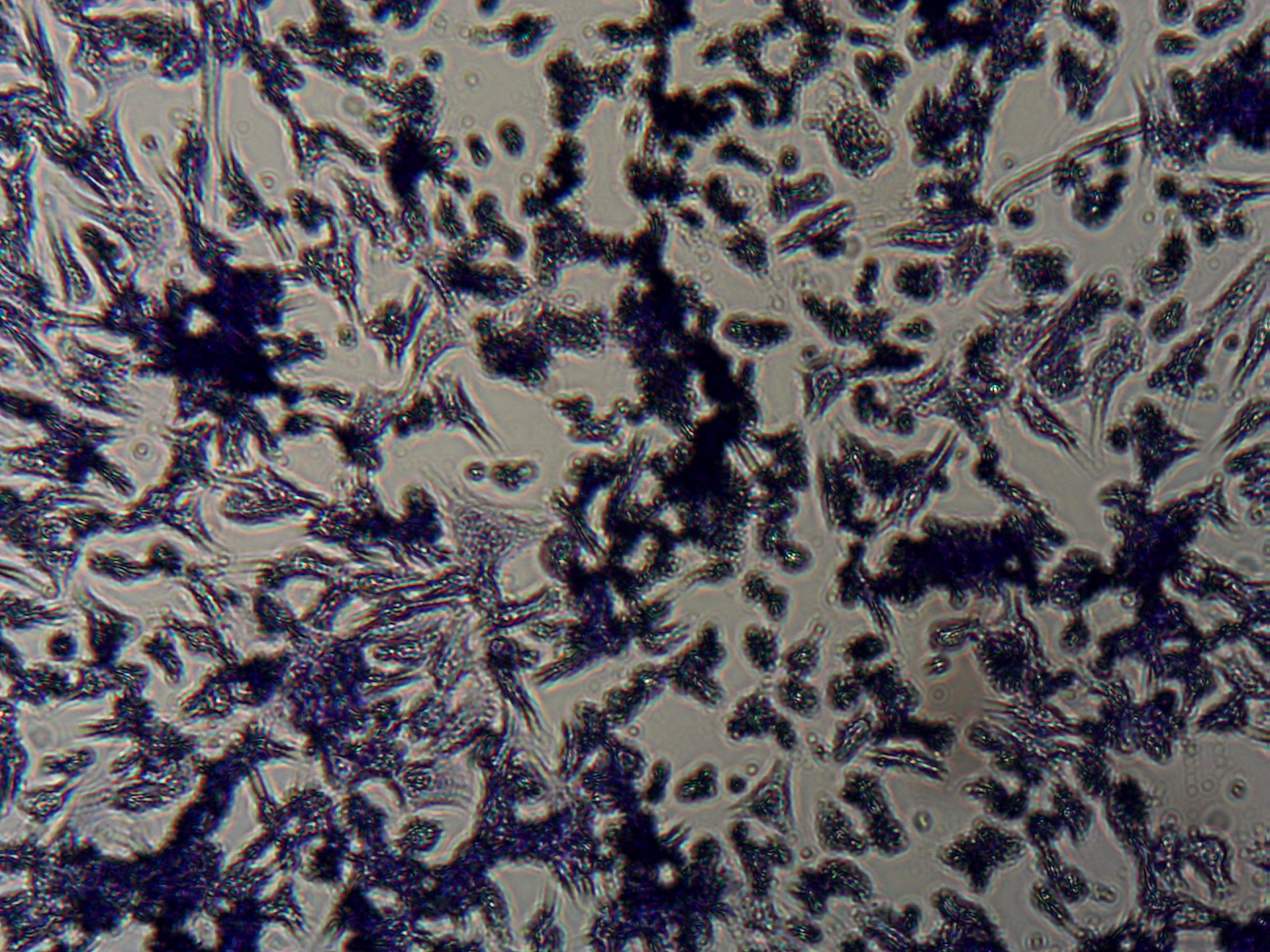

Supplement: S4 Fig — (JPG) [file pone.0292350.s007.jpg]

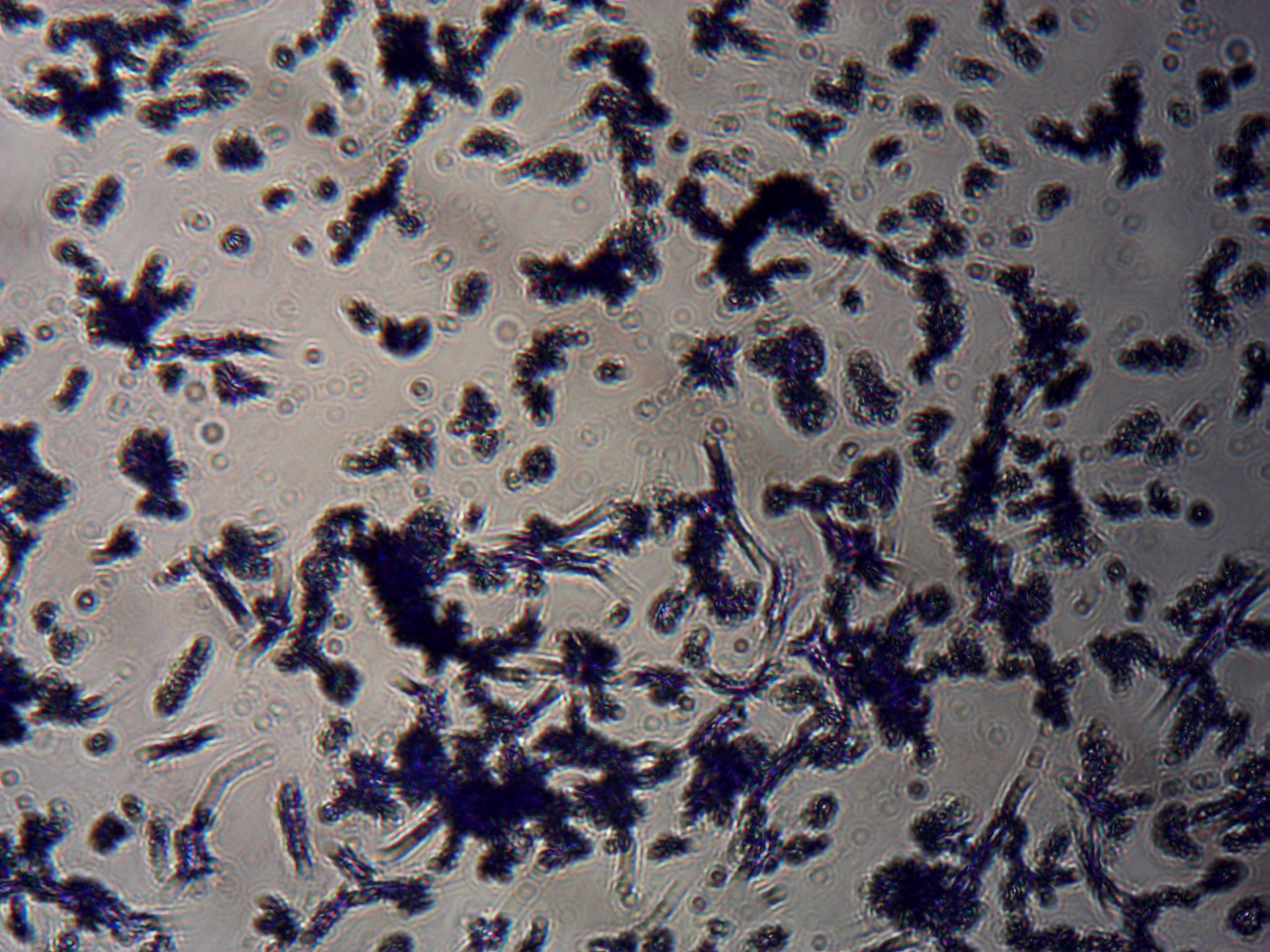

Supplement: S5 Fig — (JPG) [file pone.0292350.s008.jpg]
